# *HAWAIIAN SKIRT* controls size and floral organ number by modulating *CUC1* and *CUC2* expression

**DOI:** 10.1371/journal.pone.0185106

**Published:** 2017-09-21

**Authors:** Zinnia H. González-Carranza, Xuebin Zhang, Janny L. Peters, Veronique Boltz, Judit Szecsi, Mohammed Bendahmane, Jeremy A. Roberts

**Affiliations:** 1 Plant and Crop Sciences Division, School of Biosciences, University of Nottingham, Sutton Bonington Campus, Loughborough, Leicestershire, United Kingdom; 2 Department of Molecular Plant Physiology, Institute for Water and Wetland Research, Radboud University Nijmegen, Nijmegen, The Netherlands; 3 Laboratoire Reproduction et Développement des Plantes, Univesité de Lyon, Ecole Normale Supérieure de Lyon, Lyon, France; Instituto de Biologia Molecular y Celular de Plantas, SPAIN

## Abstract

The *Arabidopsis thaliana* F-box gene *HAWAIIAN SKIRT* (*HWS*) affects organ growth and the timing of floral organ abscission. The loss-of-function *hws-1* mutant exhibits fused sepals and increased organ size. To understand the molecular mechanisms of *HWS* during plant development, we mutagenized *hws-1* seeds with ethylmethylsulphonate (EMS) and screened for mutations suppressing *hws-1* associated phenotypes. We isolated the *shs1/hws-1* (*s**uppressor of* *h**w**s**-1*) mutant in which *hws-1* sepal fusion phenotype was suppressed. The *shs1/hws-1* mutant carries a G→A nucleotide substitution in the *MIR164* binding site of *CUP-SHAPED COTYLEDON 1* (*CUC1*) mRNA. *CUC1* and *CUP-SHAPED COTYLEDON 2* (*CUC2*) transcript levels were altered in *shs1*, renamed *cuc1-1D*, and in *hws-1* mutant. Genetic interaction analyses using single, double and triple mutants of *cuc1-1D*, *cuc2-1D* (a *CUC2* mutant similar to *cuc1-1D*), and *hws-1*, demonstrate that *HWS*, *CUC1* and *CUC2* act together to control floral organ number. Loss of function of *HWS* is associated with larger petal size due to alterations in cell proliferation and mitotic growth, a role shared with the *CUC1* gene.

## Introduction

Understanding how organ growth is controlled is a pivotal step to manipulating crop yield through the production of bigger and more robust plants. The formation of mature organs requires coordinated regulation of cell proliferation and expansion [1, 2]; the regulatory pathways of lateral organ growth modulated by cell number and size has been reviewed [3].

A recent meta-analysis of growth parameters of leaves and roots of Gramineae and Eudicotyledonous, demonstrated that cell proliferation rather than cell expansion determines the final size of plant organs; the number of dividing cells rather than the rate of division determines cell production; smaller cells leaving the meristem elongate more than bigger cells; and the differences in mature cell size are determined by cell expansion duration [4].

Flowers are the reproductive units of a plant, understanding the regulatory mechanisms that form and shape them is paramount. Flowers arise from the undifferentiated stem cells of the floral apical meristem [5]; a mature *Arabidopsis thaliana* flower has four sepals, four petals, six stamens and two congenital fused carpels that form the gynoecium; these organs emerge from four concentric whorls and their number is highly conserved [6]. Complex regulatory pathways of homeotic and cadastral genes determine floral organ identity, morphology, position, size and number in an Arabidopsis flower [7]. Cadastral genes prevent organ fusion by defining expression boundaries of floral identity genes [8, 9]. The boundaries are shaped by a combination of a reduction in the frequency of cell division, a cessation of DNA synthesis, and an interruption in the expression of cell cycle related genes in a group of specialized cells forming the boundaries [10, 11].

Many genes defining floral whorls, affecting boundary formation and floral organ number have been described (for reviews see [12–14]). Among these genes, *CUP SHAPED COTYLEDON1* and *2* (*CUC1* and *CUC2*) NAC transcription factors, are essential for boundary formation and promotion of *SHOOT MERISTEMLESS* (*STM*) expression to initiate the shoot meristem [15–19].

*MIR164* regulate boundary formation and floral organ number by establishing and maintaining the boundary domain by controlling post-transcriptional degradation of the *CUC1* and *CUC2* mRNAs [20–22]. The *early extra petals1* (*eep1*), a mutant of *MIR164C*, has increased petal number in the youngest flowers [20]. Constitutively expressing the *MIR164B* gene results in plants showing partial fusion of cotyledons and floral organs comparable to double mutant *cuc1*/*cuc2* plants. Furthermore, such plants show fused rosette leaves, and leaf-stem and stem-pedicel fusions [21, 22]. Plants containing mRNAs resistant versions of *CUC1* or *CUC2* to *MIR164* display alterations in development including increased petal numbers and reduced sepal numbers [22], increased formation of carpel margin meristems [23], alterations in location and size of boundaries [21] and enlarged vegetative and reproductive lateral organs [24].

The characteristic organ fusions of the *hws-1* mutant are similar to these observed in the *cuc1/cuc2* double mutant [25] and the *Pro*_*35S*_:*164B* lines [21, 22], while the *Pro*_*35*_:*HWS* line displays sepal separation [26]. We hypothesize that *HWS* contributes to boundary formation and regulation of organ number by indirectly altering transcripts of *MIR164*, *CUC1* and *CUC2*.

HWS forms part of a SCF (Skp, Cullin, F-box containing complex) E3 ligase that specifically binds to a target substrate destined for degradation via the 26S proteasome. Although the F-box *HAWAIIAN SKIRT (HWS)* gene is important to control organ growth and floral organ abscission timing in *Arabidopsis thaliana* [26], its mechanism of action remains unknown. To identify the protein that HWS is targeting for degradation, we mutagenized *hws-1* seeds with ethylmethylsulphonate (EMS) to screen for mutants suppressing *hws-1* associated phenotypes.

In this study, we describe the mapping and characterisation of *cuc1-1D*, in which the sepal fusion phenotype of *hws-1* is suppressed. *cuc1-1D* is mutated in the *MIR164* target site of *CUC1* mRNA. Our data reveal that *HWS* controls floral organ number by modulating transcript accumulation levels of *MIR164*, *CUC1* and *CUC2* genes. We have also shown that *HWS* regulates cell proliferation and mitotic growth in Arabidopsis petals.

## Materials and methods

### Plant material

We obtained Arabidopsis Columbia-0 seeds from the Nottingham Arabidopsis Stock Center. Single, double and triple mutants between *hws-1*, *cuc1-1D* and *cuc2-1D* were generated by crossing the genotypes as described in [27]. The *ffo1* mutant (Landsberg background) [28] was sourced from Elliot Meyerowitz, and the *cuc2-1D* mutant (Columbia-0 background) [24] from John Walker. The F1 plants were self-pollinated and homozygous F2 lines were identified using PCR. All lines were grown in a growth room with temperature of 22±2°C, and photoperiod of 22h light/2h darkness supplemented with fluorescent lights at a light intensity of 200 μmol m^-2^s^-1^ (Polylox XK 58W G-E 93331). The *hws-1* EMS populations were maintained in a greenhouse, temperature: 23±2°C and photoperiod: 16h light/8h darkness.

To identify the nature of the mutation in allele *hws-5* (*ffo1)*, genomic DNA from seedlings from the *ffo1* mutant was extracted (Qiagen, DNAeasy Plant Mini kit) and used to amplify the genomic region from the *HWS* gene using the primers At3g61590ForcDNA and At3g61590rev. PCR reactions were performed using Platinum^®^ pfx DNA polymerase (Invitrogen). The amplified band was gel purified using Genelute™ Gel extraction kit (Sigma), and sequenced using primers At3g61590ForcDNA, At3g61590Rev, SSLPHSFor, SSLPHSRev, HS 5’endutrfor and HSmap3rev.

### EMS mutagenesis of *hws-1* and suppressor screening

Approximately 5000 seeds from the *hws-1* mutant (Columbia-0 background) were treated with EMS as described by [29] and [30]. 269 pots of 13 cm with 10–20 M1 seeds each were sown and their seeds were collected in bulked M2 families. 308 plants per population were grown individually in 1cmX1cm pots and once flowering was initiated, potential suppressors of the sepal fusion phenotype from *hws-1* were identified and isolated. Mutant lines were backcrossed four times with wild-type Columbia-0. The *cuc1-1D* homozygous line was identified by segregating away the *hws-1* mutation, by growing independent lines and by ensuring that all plants displayed extra floral organs and none were *hws-1* mutants (5 generations of plants). All putative suppressors were tested by PCR to confirm that they were in the *hws-1* background using the primers SSLPHSFor /SSLPHSRev; an Extract-N-Amp PCR kit (Sigma) was used to extract genomic DNA.

The F1 progeny from a cross between the *shs1* (*shs1/hws-1*) mutant and the *hws-5* (*ffo1*) allele was allowed to self to generate the F2 mapping population. About 500 F2 plants were grown and DNA was extracted (Sigma-Aldrich, GenElute™ Plant Genomic DNA Miniprep Kit) from those that displayed a *hws-1* like phenotype. Because the mutant allele of *shs1* is dominant in a *hws-1* mutant background, individuals with the wild type allele of *shs1* had to be selected for the map-based cloning procedure. Hence, 94 out of 500 F2 individuals with the *hws-1* mutant phenotype were selected.

Mapping analysis included a combination of InDels and SSLP markers [31]. When the region was narrowed to a 0.875MB segment, candidate genes in this region were identified and a 1.565kb genomic region of the *At3g15170* (*CUC1*) gene was sequenced.

### Plasmid constructions and plant transformation

For the complementation construct, a genomic region of the *CUC1* gene of 2.498kb containing 1.386kb promoter upstream of the ATG plus 5’ and 3’ untranslated regions, introns and exons and including the substitution mutation identified in the *cuc1-1D* allele, was amplified from *shs1/hws-1* using the primers CUC1prFor and CUC1Rev. Restriction sites were added to clone the genomic region into the PBI101.2 vector.

For the silent version construct (referenced as *CUC1-SV*), the equivalent genomic region was amplified from wild type plants and mutagenized using the QUikChange II XL site-Directed mutagenesis kit (Agilent Technologies) to introduce a silent single point mutation C→T at 1.236kb from the ATG of *CUC1*. *Escherichia coli* XL10- Gold Ultra competent cells were transformed, positive colonies were selected by PCR, and successful mutagenesis was confirmed by sequencing. The plasmid was electroporated into *Agrobacterium tumefaciens* C58 strain and Arabidopsis plants were transformed using the floral dip method described by [32].

For the *MIR164B* modified version, genomic DNA from Columbia-0 seedlings was used to amplify a region of 1.340kb comprising the *MIR164B* gene, using the primers Comp164bFor and Comp164bRev. The PCR reaction was performed using Platinum^®^ pfx DNA polymerase (Invitrogen). The obtained band was sequenced and a product containing the *Bam*HI and *Sac*I restriction sites was generated using the primers Comp164bForBamHI and Comp164bRevSacI. This segment was sub-cloned in a MOG402 engineered vector comprising two copies of the CaMV 35S promoter [26]. A single nucleotide change was introduced in nucleotide number 9 (from the 5’ end) of the binding site from C →T, so that the new version had a restored binding affinity to the mRNA of the *cuc1-1D* mutant. The plasmid was mutagenized as described previously for the *CUC1*-SV. Twenty-four independent transformants from each construct were analysed.

### Floral organs numbers, petal cell size measurements and statistical analyses

Five flowers from six plants (n = 30) stage 15a [6] were dissected. Floral organs were analyzed and counted using a Zeiss Stemi-SV6 stereomicroscope. Photographs of flowers (10 days after anthesis) were taken using a Canon Power Shot-A620 camera and captured with Canon ZoomBrowserEX5.5.0.190. Petal size and cell number were determined as described in [33] using twenty flowers from four independent plants of each genotype grown under the same conditions.

For fluorescence-activated cell sorting (FACS) analysis, 2 young leaves, 50 flowers or 200 petals from 5 independent plants were used for each genotype. Nuclei isolation and FACS analysis were performed as described in [34] using MACSQuant VYB (Miltenyi Biotec) cytometer.

Statistical analyses were undertaken and graphics created for all measurements. Regression analyses and ANOVA using generalized linear models were performed using GenStat 15.1.0.8035. Graphs were created using Microsoft Excel 2010 and annotated in Adobe Photoshop 7.0.1.

### DAPI staining

Arabidopsis flowers from Columbia-0, *hws-1*, *cuc1-1D*, *hws-1/cuc1-1D* were harvested in water containing 0.05% (v/v) Triton X-100 (Sigma). Nuclei were stained by adding 1μg/ml DAPI (Sigma) to the solution for 15 min at RT. Samples were washed twice with distilled water containing 0.05% (v/v) Triton X-100 (Sigma). Petals were dissected and DAPI staining was observed using Nikon Optiphot-2 microscope equipped with a Leica DFC320 camera.

### RT-qPCR analyses of gene expression

Total RNA from a cluster of buds and young flowers (up to stage 12, [6]) from Columbia-0, *hws-1*, *cuc1-1D*, *hws-1/cuc1-1D*, *cuc2-1D* mutants and *Pro*_*35*_:*HWS* line was extracted using TRIzol reagent (Life Technologies). Three biological replicates and two technical replicates from each sample were used to perform the RT-qPCR analyses.

Expression analyses were determined using the SuperScript III Reverse transcriptase kit (Invitrogen). The First-strand cDNA was synthesised in a 20 μL reaction containing 2μg of total RNA, 1 μL mL^-1^ oligo dT (500 ng μl^-1^) and 2 μL 5mM dNTPs. Each sample was diluted by eight fold. Standards were prepared pooling 65 μL of each sample to dilutions 1, 1:4, 1:16, 1:64 and 1:256. Three biological and two technical replicates were used per sample (n = 3).

qPCR reactions were performed using Sensimix SYBR Hi-Rox Kit (Bioline) in a final volume of 10 μL, using a Roche Light Cycler 480 II. Transcripts were quantified using the Light Cyler 480 software version 1.5 using *TUBULIN 4* (*At5g44340*) gene as the endogenous reference. Graphs were created in Excel using the data generated from the Light Cycler 480 software.

All primers described in material and methods are listed in S1 Table.

### Accession numbers

Sequence data can be found in the Arabidopsis Genome Initiative or GenBank/EMBL databases under the following accession numbers: *HWS*, *At3g61590*; *CUC1*, *At3g15170*; *CUC2*, *AT5G53950; MIR164A*, *At2g47585; MIR164B*, *At5g01747*; and *MIR164C*, *At5g27807*.

## Results

### The mutant *shs1* in *hws-1* suppresses the sepal fusion phenotype and carries a transition in the *MIR164* binding domain of *CUC1*

**Fig 1:**
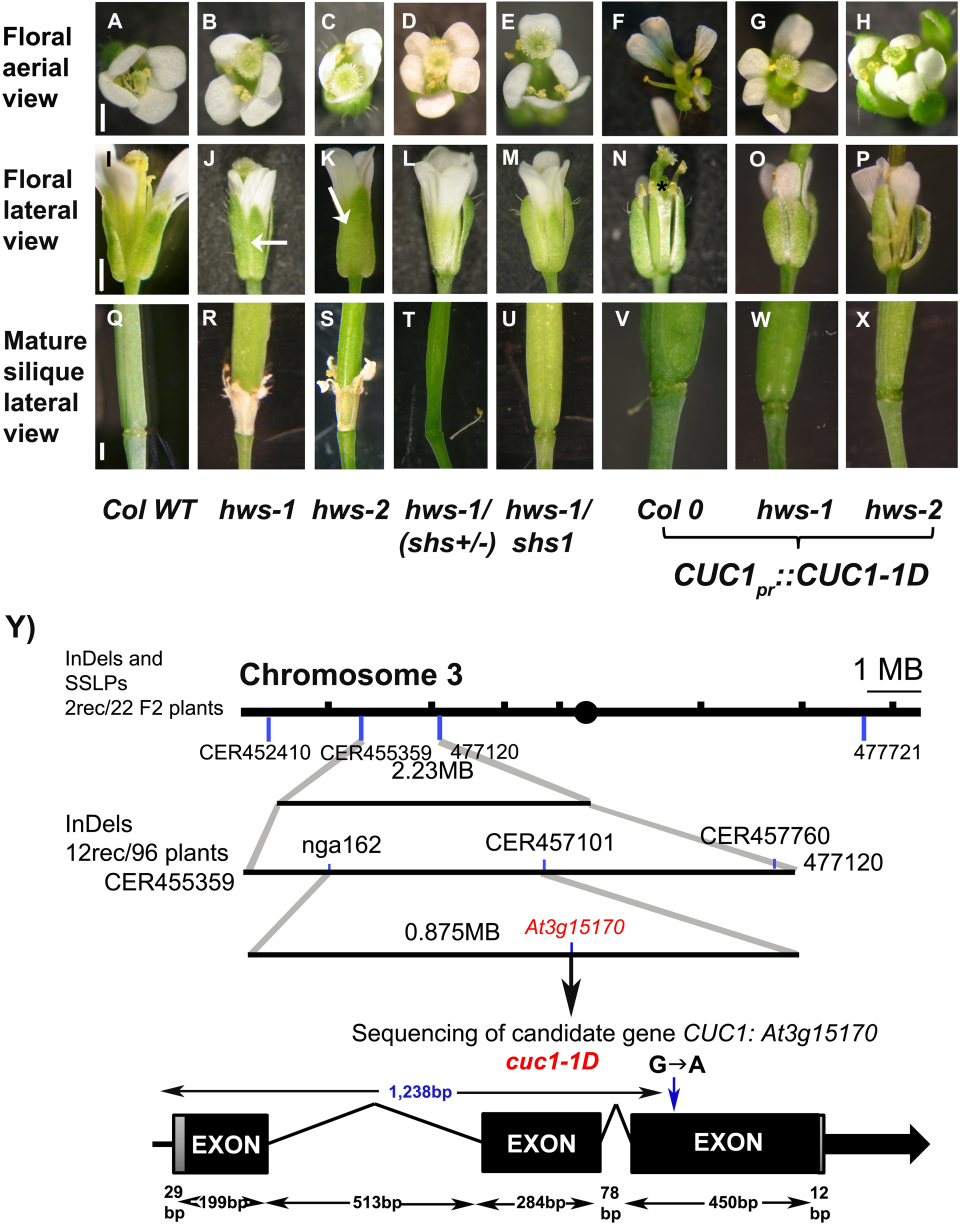
The *shs1* mutant is an allele of *CUC1*. (**A-H**), Aerial and (**I-P**), lateral views of flowers at stage 15a; and (**Q-X**), lateral view of mature green siliques. From: (**A, I, Q**), Columbia-0; (**B, J, R**), *hws-1* (Columbia-0 background);(**C, K, S**), *hws-2* (*L*er background); (**D, L, T**), *hws-1/shs*+/-; (E, M. U), *hws-1/shs1 (hws-1/cuc1-1D)*; and primary transformants of (**F, N, V**), Columbia-0; (**G, O, W**), *hws-1*; and (**H, P, X**), *hws-2* complemented with a genomic region containing the *CUC1pr*::*CUC1-1D* gene. Scale bars: 1mm. Arrows show the sepal fusions. A petal in F and a sepal on P have been removed. * in N shows stamen fusion. (**Y**), Mapping strategy used to identify the *cuc1-1D* mutation. Structure of the gene and location of the transition substitution (G→A) 1,238bp from the ATG are included, intragenic regions are represented by thin lines and exons by black boxes.

To further understand the molecular mechanism of *HWS* in plant development, we mutagenized *hws-1* seeds (Columbia background) with ethylmethylsulphonate (EMS) and screened for revertants of the *hws-1* floral sepal fusion phenotype. About 5,000 M1 plants were produced and their seeds were pooled in 269 bulked M2 families; 308 individuals of each M2 population were screened for reversion of the sepal fusion phenotype. We isolated the mutant *s**uppressor of* *h**w**s**-1* (*shs1*) in the *hws-1* background that exhibited no sepal fusion, suggesting suppression of the *hws-1* phenotype. The suppressor *shs1/hws-1* (Columbia-0) was crossed to *hws-5* (*ffo1*, *Ler* background; S1 Fig) and all the F1 progeny showed the *shs1/hws-1* mutant phenotype, indicating that the *shs1* mutation is dominant. Selfing this F1 revealed a 3:1 segregation of *shs1/hws-1*: *hws-1* in F2 (data not shown) confirming that the mutation in the *shs1/hws-1* line is a dominant allele in the *hws-1* background (Fig 1A, 1B, 1C, 1D, 1E, 1I, 1K, 1L, 1M, 1Q, 1R, 1S, 1T and 1U). This F2 progeny was used as a mapping population.

**Fig 2:**
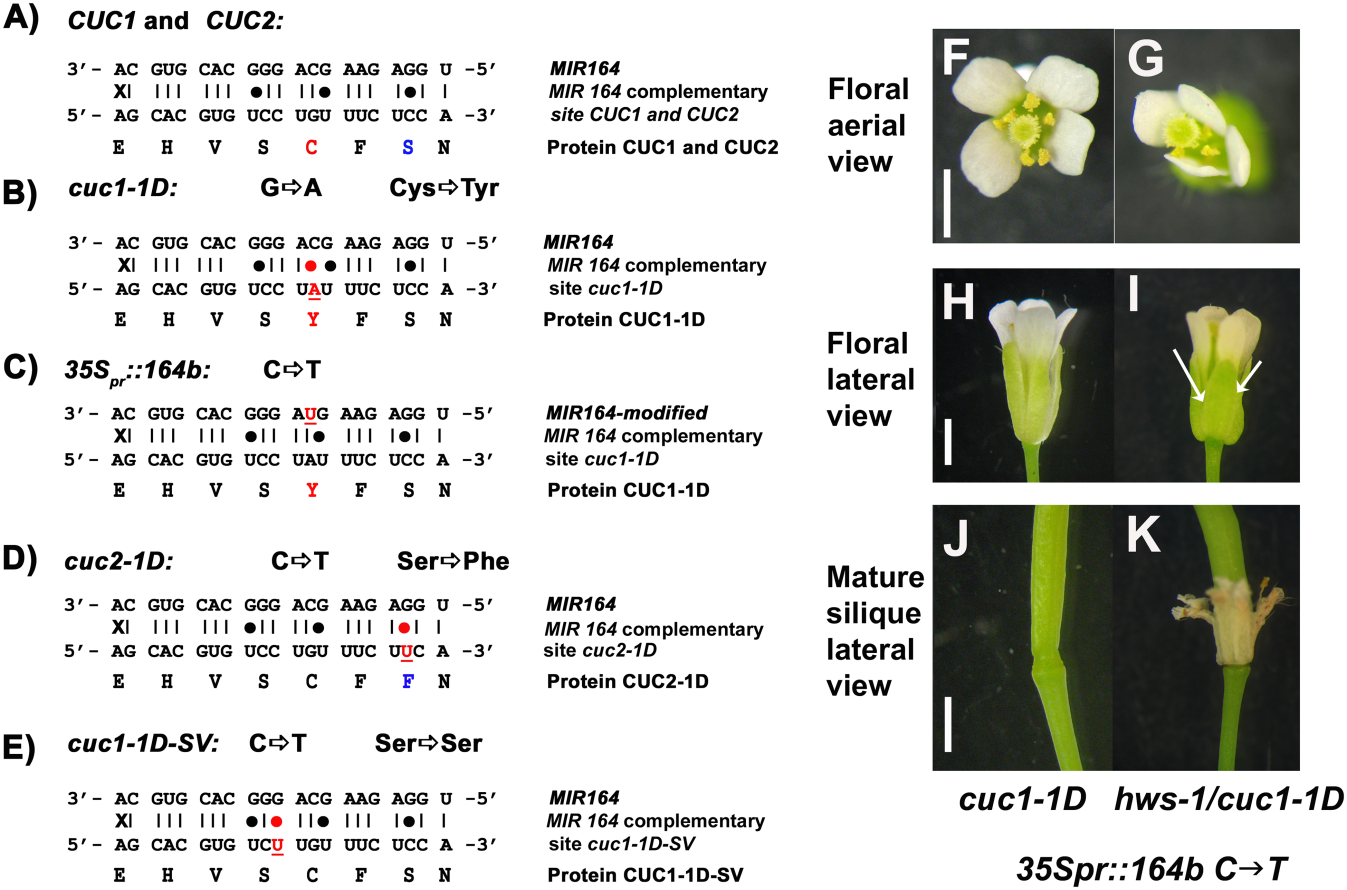
Mutations and constructs in *CUC1*, *CUC2* and *MIR164*. Schematic diagram of *MIR164*, *MIR164* complementary binding sites in *CUC1* and *CUC2* mRNAs and CUC1, CUC2 proteins or their equivalent in generated constructs; (**A**), wild type (**B**), *cuc1-1D* mutation; (**C**), *cuc1-1D* mutation and *MIR164* modified site introduced for complementation analyses; (**D**), *cuc2-1D* mutation (modified from [24]); (**E**), *cuc1-1D* silent version (*cuc1-1D-SV*). Mutations are underlined, the amino acid substitutions are identified in red/blue font, and changes in binding affinity from the *MIR164* are indicated with a red dot. (**F-K**), Complementation analyses in primary transformants using a modified version of *MIR164B*; (**F-G**), aerial and (**H-I**), lateral view of flowers at stage 15a and (**J-K**), lateral view of mature siliques from complementation lines in *cuc1-1D* and *hws-1/cuc1-1D* backgrounds using the *35S*_*pro*_::*164B C→T* construct, arrows show sepal fusion. Twenty-four primary independent transformants from each line were analysed. All transformants reverted or not the sepal fusion phenotype in the *cuc1-1D* and *hws-1/cuc1-1D* backgrounds respectively. Scale bars: 30 μm F-G and 1mm in H-K.

**Fig 3:**
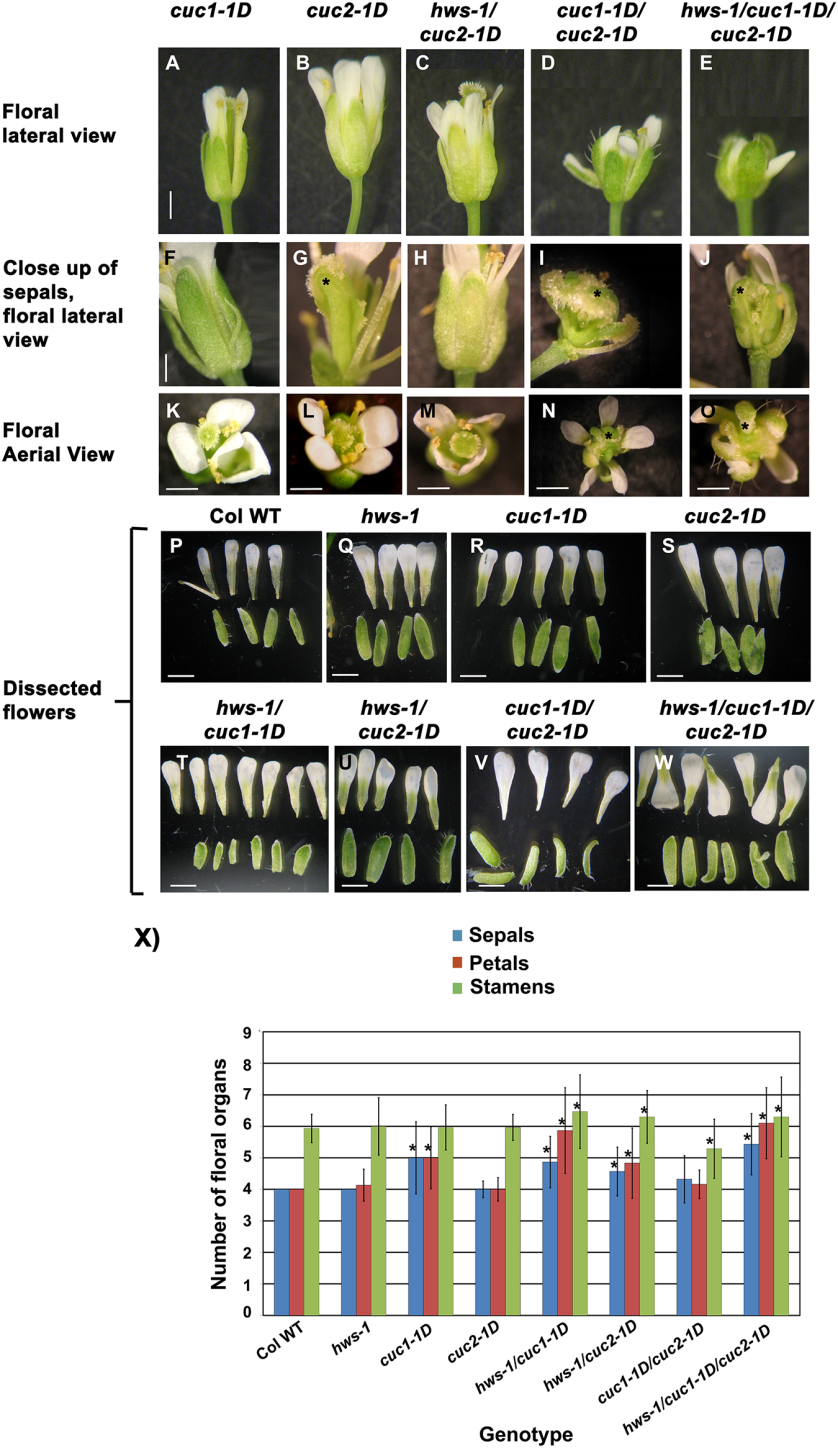
Floral organ number is affected in single, double and triple mutants of *hws-1*, *cuc1-1D* and *cuc2-1D*. Comparative phenotypic analyses of flowers at developmental stage 15a. (**A-E**), lateral view of flowers; (**F-J**), close up of sepal separation; (**K-O**), aerial view at stage 15a from: (**A, F, K**) *cuc1-1D*; (**B, G, L**), *cuc2-1D*; (**C, H, M**), *hws-1/cuc2-1D*; (**D, I, N**), *cuc1-1D/ cuc2-1D* and (**E, J, O**), *hws-1/cuc1-1D/cuc2-1D*. (**P-W**), dissected flowers at stage 15a from: (**P**) Columbia-0, (**Q**) *hws-1*, (**R**) *cuc1-1D*, (**S**) *cuc2-1D*, (**T**) *hws-1/cuc1-1D*, (**U**) *hws-1/cuc2-1D*, (**V**) *cuc1-1D/ cuc2-1D* and (**W**) *hws-1/cuc1-1D/cuc2-1D*. Scale bars: 1 mm in (**A-J**) and 300 μm in (**K-W**), * show misshapen organs. (**X**), Five flowers from six plants of each genotype were dissected and their floral organs quantified and statistically analysed by regression analyses using generalized linear models. Stars indicate a significant difference in the mean at P≤0.05 n = 30. Bars indicate SD.

A map-based cloning approach combining InDels and SSLP markers [31] located the *shs1* mutation in a 0.875 MB region at the top of chromosome 3 (Fig 1Y). This region contains approximately 1,100 genes, including *At3g15170* (*CUC1*). We analysed the genomic DNA region corresponding to *CUC1* as this provided a strong putative candidate based on similarity of phenotypes between the double *cuc1/cuc2* and the *hws-1* mutants. Sequencing of this locus in the *shs1/hws-1* mutant identified a transition mutation G→A, 1.238kb downstream of the ATG of *CUC1*. This mutation is located in the binding domain of the *MIR164* target site of *CUC1* [21, 25] and introduces the amino-acid substitution cysteine→ tyrosine in CUC1 (Fig 2A and 2B). Consequently, the *shs1* mutant was renamed *cuc1-1D*. The double mutant *hws-1/cuc1-1D* was backcrossed with Columbia-0 to obtain a *cuc1-1D* single mutant for successive analyses which displayed sepal separation (Fig 3A and 3F).

To confirm that the mutation identified in *CUC1* was responsible for the *shs1* phenotype, Columbia-0, *hws-1* and *hws-2* (T-DNA insertion; S1 Fig) plants were transformed with a 2.498kb genomic segment of the *CUC1* gene containing a 1.386kb promoter region upstream of the ATG, 5’ and 3’ untranslated regions, introns and exons and included the mutation identified in the *cuc1-1D* allele. This segment was sufficient to suppress the sepal fusion phenotypes in *hws-1* and *hws-2* mutants (Fig 1O, 1P, 1W and 1X). These data support the assertion that the single point mutation in the binding domain of *MIR164* is sufficient to suppress the floral fusion phenotype of *hws* mutants.

### A mutated version of the *MIR164B* gene reverts the floral phenotypes in *cuc1-1D* and *hws-1/cuc1-1D* backgrounds

To investigate if the phenotypes observed in the *cuc1-1D* and *hws-1/cuc1-1D* mutants result from altering the binding affinity of *MIR164* to the mRNA of *CUC1*, we generated a construct containing a 1.340kb genomic region of *MIR164B* gene expressed under the control of the CaMV 35S promoter. A point mutation was introduced to change the nucleotide C→T in the binding module, so that it matched the target in *cuc1*-1D exactly (Fig 2C). Transformation of either *cuc1-1D* or *hws-1*/ *cuc1-1D* with this modified *MIR164* transgene confirmed that this point mutation was enough to restore the wild-type and *hws-1* phenotypes of the *cuc1-1D* and *hws-1/cuc1-1D* mutants, respectively (Fig 2F, 2G, 2H, 2I, 2J and 2K); twenty-four independent transformants were analysed, all plants reverted to wild-type in *cuc1-1D* or *hws-1* in *hws-1/cuc1-1D* mutants. These data suggest that the mutation in *cuc1-1D* is enough to disrupt the binding of the mature *MIR164* molecule to the mRNA of *CUC1* and it generates a version of *CUC1* mRNA resistant to microRNA directed degradation.

### Introgression of the *cuc2-1D* mutation into *hws-1* suppresses the sepal fusion phenotype

The *MIR164* binding sites are identical in *CUC1* and *CUC2* (Fig 2A). To investigate if mutation in the *MIR164* binding site of *CUC2* could also suppress the sepal fusion in *hws-1*, the *hws-1* and *cuc2-1D* mutants were crossed (Figs 1B, 1J, 1R, 3B, 3G and 3L) [24]. *cuc2-1D* carries a transversion mutation (G→T) in the *CUC2* mRNA regulatory binding domain of the *MIR164* target site ([24]; Fig 2D). F2 population plants were genotyped by PCR and homozygous for *hws-1* and either heterozygous (data not shown), or homozygous for the *cuc2-1D* mutation were identified.

Both heterozygous and homozygous *cuc2-1D* were able to suppress the sepal fusion phenotype in the *hws-1* background; thus similar results to those observed with *cuc1-1D* (data shown for homozygous *hws-1/cuc2-1D*, Fig 3C, 3H and 3M).

These data show that a mutation in the regulatory binding domain of the *MIR164* target site of the *CUC2* mRNA is also able to suppress the *hws* fused sepal phenotype.

### *HWS* and *CUC1* regulate floral organ number in Arabidopsis

In addition to rescuing the sepal fusion phenotype of the *hws-1* mutant, the new *cuc1-1D* allele in a *hws-1* background (*cuc1-1D/hws-1*), displayed statistically significant increase of floral organs number when compared to Columbia-0. *cuc1-1D/hws-1* exhibited 4.87±0.82 sepals, 5.87±1.36 petals and 6.47±1.17 stamens (Figs 1E, 3T and 3X). *hws-1/cuc2-1D* mutant plants also showed statistically significant increases in sepal, petal and stamens number of 4.57±0.77, 4.83±1.12 and 6.3±0.84, respectively, compared to the Columbia-0 (Fig 3U and 3X). Floral organ numbers were also investigated in *hws-1*, *cuc1-1D*, *cuc2-1D*, *cuc1-1D*/*cuc2-1D* and *hws-1*/*cuc1-1D*/*cuc2-1D* mutants. The *hws-1* and *cuc2-1D* mutants showed wild-type flower organ number (Figs 1B, 3Q, 3L, 3S and 3X). The *cuc1-1D* mutant, in Columbia-0 background, had a statistically significant increase in sepal and petal numbers of 5±1.14 and 5±0.98, respectively, but not of stamens when compared to Columbia-0 (Fig 3K, 3R and 3X). A statistically significant decrease stamens number (5.29±0.94) was observed in *cuc1-1D*/*cuc2-1D* mutants when compared to Columbia-0 (Fig 3N, 3V and 3X). No statistically significant increase of petals and sepals was observed. In the *hws-1*/*cuc1-1D*/*cuc2-1D* triple mutant a statistically significant increase in the number of sepals, petals and stamens of 5.43±0.97, 6.1±1.25, and 6.3±1.26, respectively, was observed compared to the Columbia-0 (Fig 3O, 3W and 3X). We previously demonstrated that the *Pro*_*35S*_:*HWS* line displays 4 sepals, 4 petals and 6 stamens [26].

Interestingly, extra floral organs were also observed in the *hws-1* and *hws-2* plants complemented with the construct containing the *cuc1-1D* mutation, similar to *shs1/hws-1* line identified in our EMS studies (Fig 1G and 1H). However, plants harboring *cuc1-1D* mutation in Columbia-0 background, exhibited no defects in floral organ number, but floral organ shape and size were altered (Fig 1F and 1N).

These data show that adding *hws-1* to either *cuc1-1D* or cuc2-*1D* leads to increase in floral organ number. These results suggest that HWS together with CUC1 and CUC2 plays a role in regulating floral organ number in Arabidopsis.

### The binding affinity of *MIR164* to the *CUC1* mRNA and the presence of *HWS* are crucial for sepal fusion rescue and for regulating floral organ number and identity

**Fig 4:**
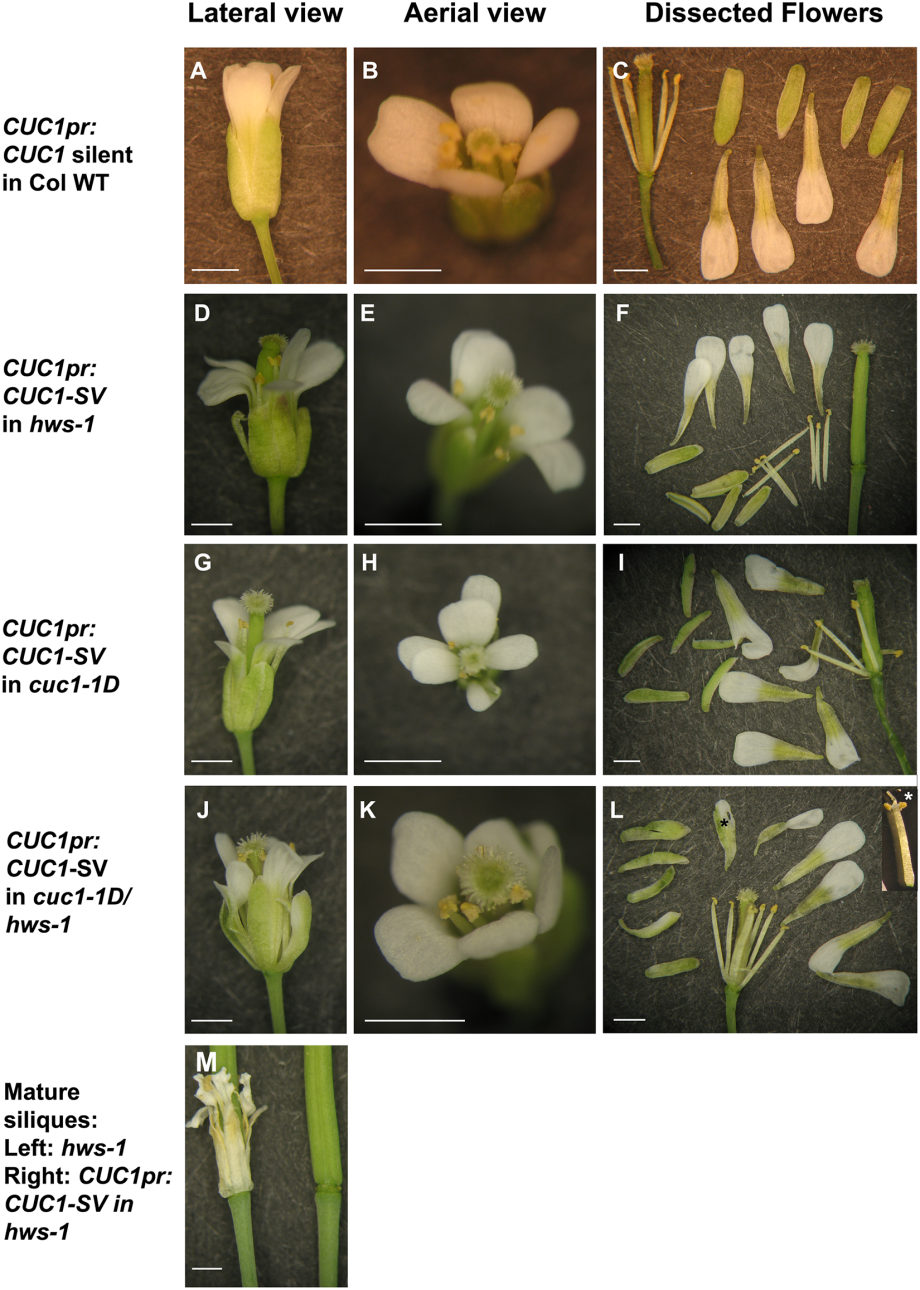
A silent mutation in *CUC1* does not change floral organ numbers in a Columbia-0 background but induces extra floral organs in *hws-1*, *cuc1-1D* and *hws-1/cuc1-1D*. (**A, D, G, J, M**), Lateral view; (**B, E, H, K**), Aerial view; (**C, F, I, L**), dissected flowers of primary transformants in the following backgrounds: (**A-C**), Columbia-0; (**D-F**), *hws-1*; (**G-I**), *cuc1-1D*; and (**J-L**), *hws-1/cuc1-1D*, note bifurcated anther inidicated with a white star in panel L. (**M**), Mature siliques showing suppression of sepal fusion in *hws-1*: left silique originated from a *hws-1* mutant, right silique originated from a primary transformant *hws-1* plant transformed with *CUC1-SV*. Scale bars: 1mm. Black and white stars show altered floral organs.

To investigate if the sepal fusion rescue and the extra floral organs phenotypes observed in the *cuc1-1D*/*hws-1* mutant is due to altering the binding affinity of *MIR164* to the mRNA of *CUC1*; or to the amino-acid substitution (Fig 2B) in the CUC1 protein; a silent mutation C→T was introduced 1.236kb downstream from the ATG of *CUC1* where *MIR164* binds. This modification does not change the amino-acid sequence, but likely weakens the binding affinity of the *MIR164* (Fig 2E) to its target sequence of *CUC1*. If additional floral organs seen in the *cuc1-1D* mutant are the consequence of changing the amino-acid, it is expected that a silent mutation in the *CUC1* gene would not rescue sepal fusion, nor produce extra floral organs in the *hws-1* or Columbia-0 backgrounds. If the extra floral organs are the consequence of changing the binding affinity of *MIR164* to the mRNA of *CUC1*, an increase in floral organ numbers in the *hws-1* background and an equal or more extreme phenotype in the *cuc1-1D* and *hws-1/cuc1-1D* lines are expected. Plants from Columbia-0, *hws-1*, *cuc1-1D* and *hws-1/cuc1-1D* were transformed with this construct (*CUC1-SV*), twenty-four independent transformants were analysed. Results show that the silent version of *CUC1* did not affect floral organ number in primary transformants in the Columbia-0 background (Fig 4A, 4B and 4C). We observed a similar effect in our complementation experiments of Columbia-0 with the *cuc1-1D* construct where organ number was not affected (Fig 1F, 1N and 1V), suggesting that the presence of an endogenous wild-type copy of *CUC1* is enough to maintain the usual floral organ numbers. The *CUC1-SV* supressed the sepal fusion phenotype and increased floral organ number in the *hws-1* background in a similar fashion as in the *hws-1/cuc1-1D* line (Fig 4D, 4E, 4F and 4M). Introducing *CUC1-SV* in the *cuc1-1D* and the *hws-1/cuc1-1D* backgrounds, resulted in increased floral organ number and more severe phenotypes were observed. In addition, in the *cuc1-1D* and *hws-1/cuc1-1D* lines, some petals appeared misshapen, some stamens appeared bifurcated and some organs showed sepal/petal chimera phenotype (Fig 4G, 4H, 4I, 4J, 4K and 4L). These results support the hypothesis that the rescued sepal fusion and increased number of floral organs in the *hws-1/cuc1-1D* line are due to the change in the binding affinity of *MIR164* to the mRNA of *CUC1* and not to the amino-acid substitution in the CUC1 protein.

### *HWS* is involved in the control of cell proliferation in petals of Arabidopsis

**Fig 5:**
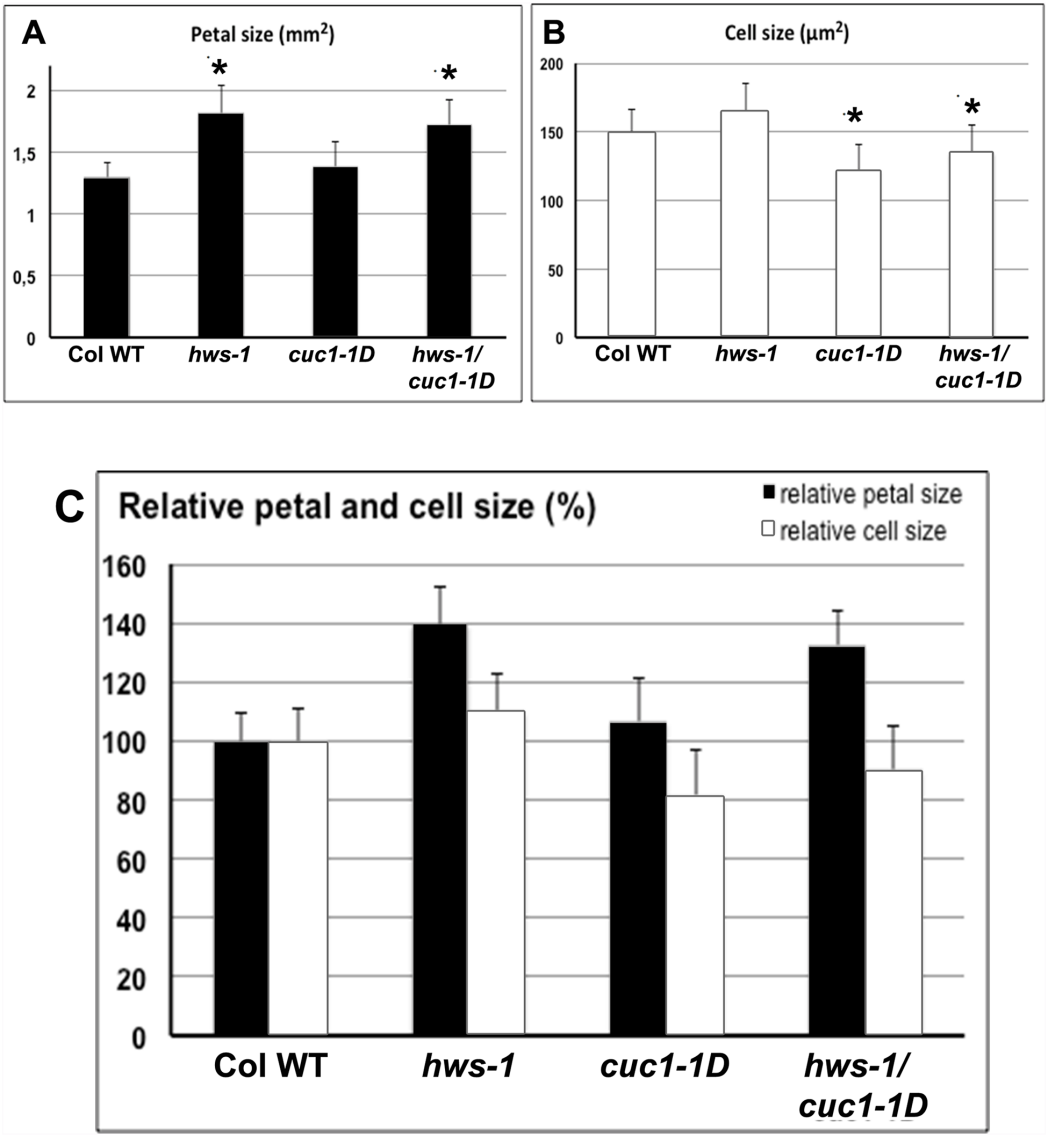
*HWS* affect cell proliferation in petals. Analyses of (**A**), petals size (mm^2^) and (**B**), petal cell size (μm^2^) in Columbia-0, *hws-1*, *cuc1-1D*, and *hws-1/cuc1-1D*. Five flowers from four independent plants from each genotype were dissected and their size and the size of petal cells were determined. (**C**), Relative petal and cell sizes compared to Columbia-0 (100%). Stars indicate a significant difference in the mean at P≤0.001 n = 80.

To investigate the difference in floral organ size observed between Columbia-0 and *hws-1*, *cuc1-1D* and *hws-1/cuc1-1D*, 80 petals from 20 flowers from each genotype were dissected to determine petal size and cell number. Compared to Columbia-0, petals of *hws-1* were about 40% bigger in size (Figs 3Q and 5A). This difference was statistically significant (p<0.001). Petal cell size measurements showed no significant difference between wild-type and *hws-1* (Fig 5B). Indicating that the increased size of *hws-1* petals is likely to be associated with increased cell number. These data suggest a direct or an indirect negative role of *HWS* on cell proliferation (Fig 5C).

Petals of *cuc1-1D* were similar to wild-type (Figs 3R, 5A and 5C). However, cell size measurement revealed that *cuc1-1D* cells were smaller than that of wild-type petals, suggesting that petals of *cuc1-1D* have more cells compared to the wild-type (Fig 5B). These data also support a role of *CUC1* in cell proliferation, thus in agreement with previously reported data [35].

The double mutant *hws-1/cuc1-1D* exhibited a statistically significant increase in petal size (about 35%) similar to that observed in *hws-1* (Fig 3T), showing that adding *cuc1*-1D mutation did not modify petal size. As mentioned above. Petal cell size in *hws-1* was not different from wild-type, but the double mutant *hws-1/cuc1-1D* showed reduction in cell size similar to that observed in *cuc1-1D* mutant (Fig 5A, 5B and 5C). DNA content measurements showed a normal ploidy level in *hws-1*, *cuc1-1D* and *hws-1/cuc1-1D* leaves, flowers and petals with fractions of cells at 2N, 4N and 8N similar to Columbia-0 (S2 Fig). *In situ* DAPI staining in petals shows the presence of only one nucleus per cell in all cell types of *hws-1*, *cuc1-1D* and *hws-1/cuc1-1D*, and this was comparable to that observed in wild-type petals (S3 Fig). These data show that there is no endoreduplication in *hws-1*, *cuc1-1D* or *hws-1/cuc1-1D* mutants and suggest that both *HWS* and *CUC1* likely negatively control cell proliferation and mitotic growth in *Arabidopsis* petals. However, more data are required to address more precisely how *HWS* and *CUC1* cooperate to regulate cell proliferation and whether they act on the duration of cell proliferation and/or the rate of cell division.

### A feedback loop mechanism between *MIR164* and *CUC* is present in flowers

The *cuc1-1D* mutation introduces a substitution (G→A) in the *CUC1 MIR164* target site (nucleotide 9/21 from the 5’ end; Fig 2B). It has been demonstrated that cleavage of *CUC1* mRNA occurs between nucleotides pairing to residue10 of the *MIR164* [36]. To investigate whether this change in the *CUC1* mRNA modifies the regulatory effectiveness of *MIR164*, transcript levels of *CUC1*, *CUC2*, *HWS1*, *MIR164A*, *MIR164B and MIR164C* genes were analysed by RT-qPCR in buds and young flowers from wild-type, *hws-1*, *Pro*_*35S*_:*HWS* [26], *cuc1-1D* (in Columbia-0 background), *hws-1/cuc1-1D* and *cuc2-1D* lines.

**Fig 6:**
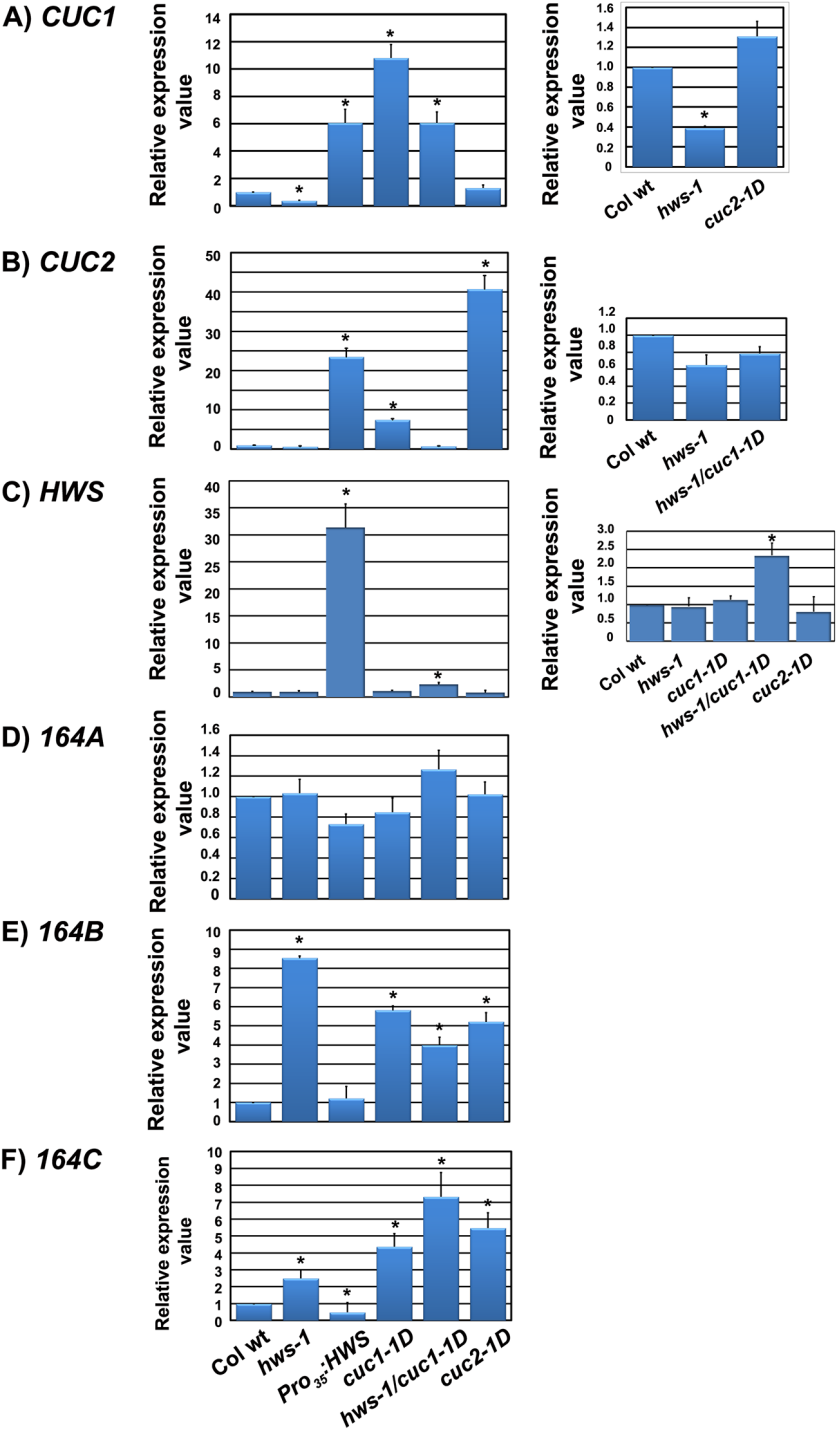
Transcript levels of *CUC1 CUC2*, *MIR164A*, *MIR164B* and *MIR164C* genes are affected in single and double mutants and in the *Pro*_*35*_:*HWS* lines. RT-qPCR measurements of (**A**), *CUC1*; (**B**), *CUC2*; (**C**), *HWS*; (**D**), *MIR164A*; (**E**), *MIR164B*; (**F**), *MIR164C* RNA levels in Columbia-0, *hws-1*, *35S*_*pro*_:*HWS*, *cuc1-1D*, *hws-1/cuc1-1D* and *cuc2-1D*. Stars indicate a significant difference in the mean at P≤0.001. Relative expression values represent the mean ± SD of three biological replicates and two technical replicates from each sample (n = 30).

Relative to the wild-type, a statistically significant over-accumulation of *CUC1* mRNA was observed in *Pro*_*35S*_:*HWS*, *hws-1/cuc1-1D* (about six fold) and in *cuc1-1D* lines (about eleven fold). Conversely, in the *hws-1* mutant line *CUC1* mRNA accumulation was significantly reduced compared to the WT control (p<0.001). No statistically significant accumulation of *CUC1* mRNA, compared to the wild-type, was observed in the *cuc2-1D* mutant (Fig 6A). *CUC2* showed statistically significant higher transcript levels in *Pro*_*35S*_:*HWS*, *cuc1-1D* and *cuc2-1D* lines, with approximately twenty, seven and forty fold over-accumulation, respectively, compared to the wild-type (p<0.001). The difference in the levels of *CUC2* mRNA in *hws-1* and *hws-1/cuc1-1D* were not statistically significant compared to the control (Fig 6B). A statistically significant over-accumulation of *HWS1* mRNA relative to wild-type of about 30 and 2.3 fold was observed in both, *Pro*_*35S*_:*HWS*, and *hws-1/cuc1-1D* lines, respectively (p<0.001). Slight differences in *HWS* transcript that were not statistically significant were observed in *hws-1*, *cuc1-1D* and *cuc2-1D* compared to the control (Fig 6C). The *hws-1* mutant harbours a 28 nucleotides deletion mutation near the 3’end (S1 Fig) thus some transcript accumulation is expected in the *hws-1* mutant line against a null mutant. These results suggest that the sepal fusion phenotype observed in the *hws-1* mutant, which phenocopies the sepal fusion of the double mutant *cuc1*/*cuc2* [25], is due to a reduction of *CUC1* transcript. Overexpression of the *HWS* gene results in an increase of transcript levels of *CUC1* and *CUC2* genes which allows sepal separation in the *Pro*_*35*_:*HWS* line.

Transcript levels of *MIR164A* were not significantly altered in any of the studied lines (Fig 6D). However, transcript levels of *MIR164B* and *MIR164C* were significantly increased by about 8 and 2, 6 and 4, 4 and 7 and 5 fold in the *hws-1*, *cuc1-1D*, *hws-1/cuc1-1D* and *cuc2-1D* mutants, respectively (p<0.001), while in the, *Pro*_*35S*_:*HWS* they were similar or lower than in the wild-type (Fig 6E and 6F). Our RT-PCR results in the *Pro*_*35S*_:*HWS* indicate that the target of HWS likely affects accumulation of *MIR164*.

In agreement with previously reported data [35], our results also show that in floral buds and flowers of our lines studied, *CUC1* and *CUC2* transcripts are regulated by *MIR164B* and *MIR164C*. It was reported that expression of *MIR164A*, *MIR164B* and *MIR164C* in inflorescences of wild type, single, double and triple *MIR* mutants show partial overlap [35]. In our material, we could not detect changes of expression of *MIR164A*, suggesting that regulation for each *MIR164* gene may be different and *MIR164B* and *MIR164C* are regulated by *HWS*.

Mutation of the *MIR164* target domain in *CUC1* or *CUC2* results in an over-accumulation of *MIRNA164B* and *MIRNA164C* transcripts, suggesting a feedback loop mechanism where higher levels of *CUC1* or *CUC2* mRNA trigger the accumulation of *MIRNA164B* and *MIRNA164C* to reduce the higher levels of *CUC* transcript. These results suggest that the sepal fusion phenotype observed in the *hws-1* mutant, which phenocopies the sepal fusion of a *MIR164B* overexpressing line [21, 22], is due to an increase of *MIR164B* and *MIR164*C levels, while overexpressing the *HWS* gene results in a decrease of transcript levels of *MIR164C*. Moreover, the data indicate that the nucleotide changed in *cuc1-1D* is crucial for the regulation of transcript levels of the *CUC1* mRNA by *MIR164*.

## Discussion

Our work here has demonstrated that *HWS* contributes to the co-ordination of floral organ number and boundary formation by altering *MIR164* and *CUC1* transcript levels. We have shown that the regulatory domain of *MIR164* is crucial for this event to take place. *HWS* and *CUC1* also regulate cell number and size in petals, and influence cell proliferation.

We generated and characterized an EMS mutant suppressor line of the *hws-1* mutant. To generate the mapping population and to identify our mutant gene, we used our discovery of a fifth mutant allele of the *HWS* gene in the previously identified *ffo1* mutant. The *ffo1* mutant was isolated from a genetic screen for modifiers of the F-box gene *UNUSUAL FLORAL ORGANS* (*UFO*) meristem activity. [28]. *UFO* controls meristem determination, organ primordia identity and cell propagation in the developing boundaries between floral organs [37].

*cuc1-1D* is a dominant mutant of *CUC1* and was identified because it suppresses the *hws-1* sepal fusion phenotype. We demonstrated that *cuc2-1D* [24] also suppresses the sepal fusion phenotype of *hws-1*, and both *hws-1/cuc1-1D* and *hws-1/cuc2-1D* have increased floral organ numbers. The *cuc1-1D* mutation has two major effects: (1) a single nucleotide change in the binding domain of *MIR164* of the *CUC1* mRNA transcript and (2) an amino-acid substitution in the CUC1 protein. Two hypotheses might account for how the *cuc1-1D* mutation leads to reversion of the sepal fusion phenotype. The amino-acid substitution in the CUC1 protein could change the conformation of the protein affecting its functionality, or alternatively, the nucleotide change could affect the binding affinity of the *MIR164* to the *CUC1* mRNA. Our results show that the sepal fusion rescue is a consequence of changing the binding affinity of *MIR164*. Moreover, it was previously reported that protein accumulation of CUC1 and CUC2 increase at boundary regions and in the center of meristems in plants containing miRNA cleavage-resistant versions of *CUC1* and *CUC2* translationally fused to GFP driven by their own promoters [35]. These published data taken together with our data, suggest that appropriate levels of *CUC* genes are necessary to maintain organ number and boundary formation in flowers. The observed phenotypes in *hws-1*, *cuc1-1D* and *hws-1*/*cuc1-1D* with either a mutated *MIR164* or a silent mutation of *CUC1* introgressed into them demonstrate that the efficacy in generating a *MIR164* resistant allele of the *CUC1* gene lies in the nucleotide change rather than the amino-acid substitution. Complementation analyses in *hws-1*, *cuc1-1D* and *hws-1*/*cuc1-1D* lines using a silent *CUC1* construct not only rescued the sepal fusion phenotype of the *hws-1* mutant, but phenocopied the increase of floral organ numbers observed in the original *hws-1*/*cuc1-1D* line. These findings support the hypothesis that correct binding of *MIR164* to the *CUC1* mRNA is important for sepal boundary formation and regulation of floral organ number. *HWS* is an F-box protein that targets for degradation a yet unidentified protein, so it is likely that its effect in floral organ number is indirect.

When a *CUC1* silent mutant version was introduced into the *cuc1-1D* and *hws-1/cuc1-1D* backgrounds, floral organ number was increased and more severe phenotypes were observed: some misshapen petals, bifurcations in stamens, and some mosaic organs between sepals and petals in the same organ. These findings suggest that increased amounts of CUC1 protein affect the homeostasis for the correct number and development of floral organs, possibly by altering the spatial expression of B function homeotic genes or by interfering with primordia formation.

The size of *cuc1-1D* mutant petals is comparable to wild-type, but their cells are smaller than wild-type. It has been proposed that *MIR164* limit the expansion of the boundary domain by degrading *CUC1* and *CUC2* mRNAs [21] and *NAC* genes repressing growth [38]. Though it has been demonstrated that *CUC1* regulates *STM* and a feedback loop exists [38, 39], it is possible that *CUC1* regulates the expression of other target genes yet to be identified. [40] reported that the role of *CUC* genes in the definition of inter-sepal boundaries is to suppress growth of sepal tissues. Our findings suggest that *HWS* and *CUC1* regulate cell size and number in petals and perhaps within the floral organ boundaries and their interaction modulate organ size.

We have shown that transcript levels of *CUC1* are down regulated in the *hws-1* mutant and both *CUC1* and *CUC2* are up regulated in the *Pro*_*35*_:*HWS* line, supporting our hypothesis that *HWS* is indirectly involved in modulating organ size and boundary formation by regulating *CUC1* and *CUC2* genes. Interestingly, in a *cuc1-1D* mutant a seven-fold increase in expression of *CUC2* can be observed, suggesting that in this particular mutant insufficient *MIR164* is produced to maintain normal transcript levels of the *CUC1* and *CUC2* genes, or there can be a sequestration of *MIR164* by the mutated *CUC1* gene.

*HWS* indirectly contributes to boundary formation by regulating *CUC1* and *CUC2*. In the *hws-1* mutant, *MIR164B* and *MIR164C* levels are elevated, while levels *MIR164A* are unchanged. Remarkably, *MIR164A* is activated in leaf primordia under continuous expression of *STM*, a direct regulator of *CUC1* and *CUC2* during SAM formation and cotyledon separation [38, 39, 41], thus suggesting the existence of different mechanisms for boundary formation in leaves and flowers.

The sepal fusion phenotype observed in the *hws-1* mutant, which phenocopies the sepal fusion of a *MIR164B* overexpression line [21, 22], is likely due to an increase of *MIR164B* and *MIR164B*C levels. Overexpressing *HWS* results in a decreased transcript level of *MIR164*, suggesting that the target of HWS play an important role in regulating *MIR164*, either in their production, function or degradation; indeed, our own work and that of [42] propose a role for *HWS* in the microRNA pathway.

Our results suggest a feedback loop mechanism where higher levels of *CUC1* or *CUC2* mRNA trigger the accumulation of *MIR164B* and *MIR164C* to reduce the higher levels of *CUC1* transcript. We propose that *HWS* regulates a target that directly or indirectly modulates *MIR164B* and *MIR164C* which, in turn, negatively regulates *CUC1* and *CUC2* to control formation of sepal boundaries and floral organ number. Alternatively, the target for *HWS* might modulate *CUC1* and *CUC2* levels, which subsequently alter *MIR164B* and *MIR164C* levels. Our results suggest that *CUC1* regulates *MIR164* directly or indirectly via a feedback loop mechanism. There is a possibility that such mechanism also exists for *CUC2*. It is likely that these feedback loop mechanisms are disrupted in the *Pro*_*35*_:*HWS* line where the target for HWS is reduced or absent.

Supporting our results, [43] reported that the poplar *HWS* orthologue (*PtaHWS*) is part of a regulatory network involving *PtaNAC1* and *PtamiRNA164e*, and proposed its importance for lateral root formation in response to low nitrogen conditions. [44] showed that *HWS* controls root meristem activity. The authors also suggested that HWS may regulate cell division in the transit amplifying cells adjacent to the quiescent centre of Arabidopsis roots.

*RABBIT EARS* (*RBE*) encodes a *SUPERMAN*-like zinc finger transcription factor that defines earlier sepal-petal whorl boundary and inter-sepal boundaries by repressing *AGAMOUS*, a keeper of spatial boundaries in Arabidopsis, and it acts in the same pathway and downstream of *UFO* [45]. [46] reported that *RBE* regulates the expression of all three *MIR164* genes, and that RBE interacts with the promoter of *MIR164C*, a gene that has been reported to be involved in the regulation of petal number in Arabidopsis [20]. Moreover, *PTL* that acts upstream of *RBE* [47] represses growth in the boundaries between sepal primordia and the outermost whorl [48]. Loss of function of the *PTL* gene results in an increase in the size of the inter-sepal zone of floral buds by 35–40% due to cell proliferation but not due to changes in cell size. It has been hypothesized that the role of PTL in the boundary region is to keep its size in check [40].

Analyses of the expression of the *PTL*, *RBE*, *AG* and *UFO* genes in our mutant collection will identify whether the role of *HWS* in floral organogenesis and boundary formation occurs via this pathway. Protein-protein interaction studies are underway to identify if any of these genes interact with HWS.

It has been reported that *CUC1* and *CUC2* genes interact with *SPATULA* (*SPT*) to control carpel margin development [49] and promote the formation of carpel margin meristem during Arabidopsis gynoecium development [23]. Our studies suggest that *HWS* may also be a key regulator gene during fruit development as the triple mutant *hws-1/cuc1-1D/cuc2-1D* showed severe reduction in seed production (data not shown). These findings may be relevant to address food security issues.

In conclusion, our findings add a new layer of complexity to the gene regulatory mechanisms that are involved controlling cell growth, organ development and boundary formation in Arabidopsis flowers.

## Supporting information

S1 Fig *ffo1* mutant is a novel allele of *HWS*. (**A**) *hws-1* mutant. (**B**), *floral fusion organs1* (*ffo1*) mutant in Landsberg erecta [28]. The *ffo1* mutant, renamed here as *hws-5*, is an allele of *HWS*. (**C**), The double mutant *ffo1*/*hws-1* exhibits fused sepal phenotype. (**D**), F1 progeny of the *shs1/hws*-1 suppressor line crossed to *ffo1* (*hws-5*) shows a *shs1/hws-1* phenotype. Side view of mature green siliques are shown. Scale bar: 1mm. (**E**), Structure of the *HWS* gene; the intragenic region in the 5’UTR is indicated as a fine line, positions of all *HWS* known alleles are indicated in this figure. We identified *hws-2* in our previous study [26]. *hws-3* and *hws-4* were identified in a suppressor screen of the *shortroot* (*shr*) mutant [43]. Sequencing analyses confirmed that *ffo1* (*hws-5*) carries a G to A mutation 630bp from the ATG resulting in a premature opal stop codon. This newly identified allele was used in the mapping analyses and positional cloning for the *hws-1* suppressor mutant. Delta symbol indicates the deletion in the *hws-1* allele. (**F**), Nucleotide and amino acid sequences of the *HWS* gene indicating the exact position of the five known *HWS* alleles; mutations are indicated in underlined capital fonts. Inverted delta indicates the site of the T-DNA insertion in the *hws-2* allele.
(TIF)

S2 Fig FACS analysis on isolated nuclei. Nuclei were isolated from young leaves, flowers and petals of *Arabidopsis thaliana* Columbia-0, *hws-1*, *cuc1-1D*, *hws-1/cuc1-1D*. After DAPI staining, samples were analysed by FACS. Peaks represent cells with 2N, 4N and 8N DNA content. The X axis represents DAPI intensity and the Y axis shows cell number.
(TIF)

S3 Fig In petal DAPI staining. (**A**), Petal of Arabidopsis thaliana. DAPI stained nuclei were observed in the top part conical cells, in the middle transition area and at the bottom part of the petal (white squares) Bar = 0.5mm. (**B-E**), DAPI stained nuclei in upper conical cells of Arabidopsis petal from Columbia-0, *hws-1*, *cuc1-1D*, *hws-1/cuc1-1D*, respectively. (**F-I**), DAPI stained nuclei in cells of the middle transition area of Arabidopsis petal from Columbia-0, *hws-1*, *cuc1-1D*, *hws-1/cuc1-1D*, respectively. (**J-M**), DAPI stained nuclei in cells of the bottom area of Arabidopsis petal from Columbia-0, *hws-1*, *cuc1-1D*, *hws-1/cuc1-1D*, respectively. Scale bar: 25μm.
(TIF)

S1 Table Sequence of primers used in this study. Experiment or procedure, primer name and sequences of primers are included.
(DOC)
