# Supplementary figures and images for: HAWAIIAN SKIRT controls size and floral organ number by modulating CUC1 and CUC2 expression
Source: PLoS One. 2017 Sep 21;12(9):e0185106. doi: 10.1371/journal.pone.0185106 (PMC5608315; doi:10.1371/journal.pone.0185106)

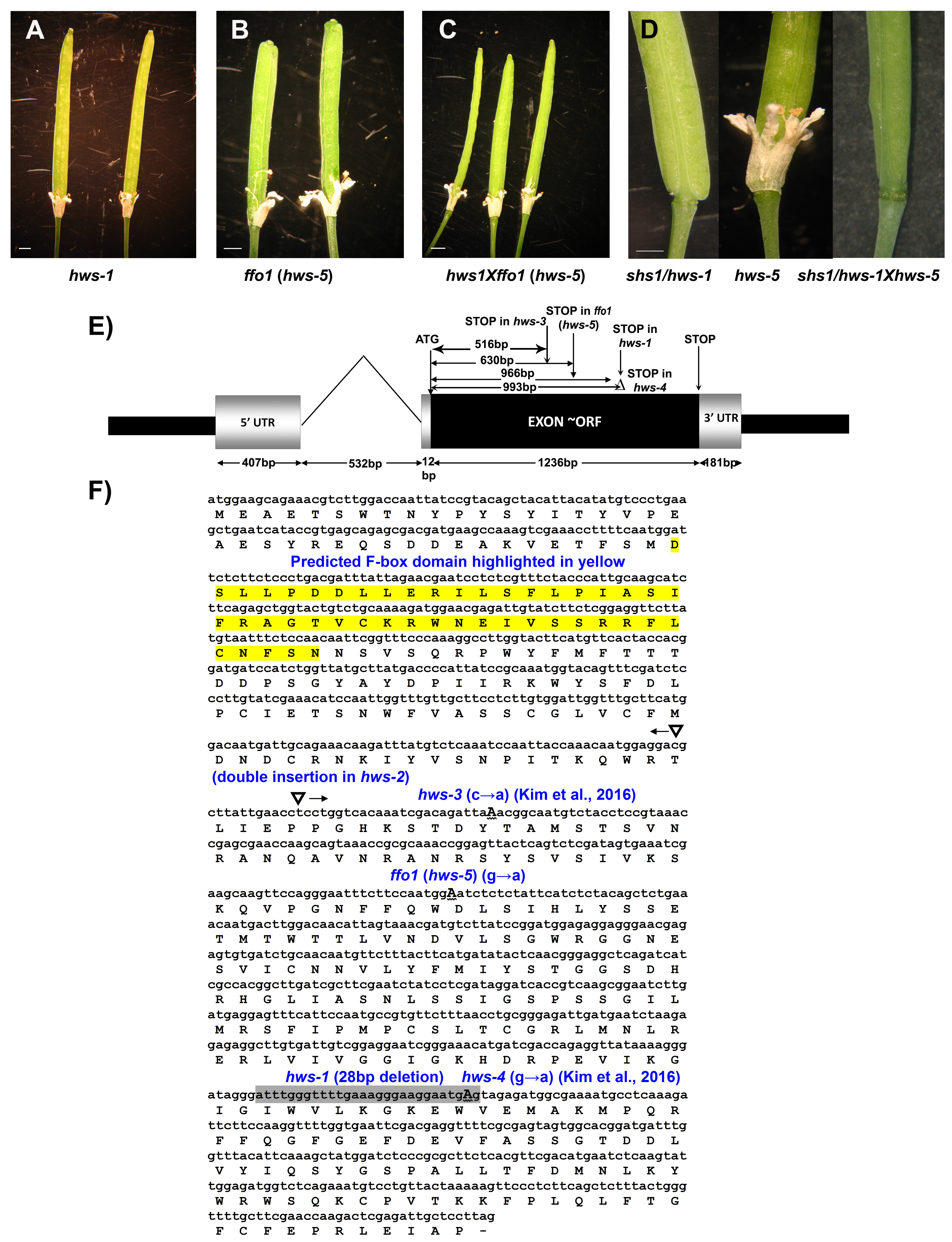

Supplement: S1 Fig — (A) hws-1 mutant. (B), floral fusion organs1 (ffo1) mutant in Landsberg erecta [28]. The ffo1 mutant, renamed here as hws-5, is an allele of HWS. (C), The double mutant ffo1/hws-1 exhibits fused sepal phenotype. (D), F1 progeny of the shs1/hws-1 suppressor line crossed to ffo1 (hws-5) shows a shs1/hws-1 phenotype. Side view of mature green siliques are shown. Scale bar: 1mm. (E), Structure of the HWS gene; the intragenic region in the 5’UTR is indicated as a fine line, positions of all HWS known alleles are indicated in this figure. We identified hws-2 in our previous study [26]. hws-3 and hws-4 were identified in a suppressor screen of the shortroot (shr) mutant [43]. Sequencing analyses confirmed that ffo1 (hws-5) carries a G to A mutation 630bp from the ATG resulting in a premature opal stop codon. This newly identified allele was used in the mapping analyses and positional cloning for the hws-1 suppressor mutant. Delta symbol indicates the deletion in the hws-1 allele. (F), Nucleotide and amino acid sequences of the HWS gene indicating the exact position of the five known HWS alleles; mutations are indicated in underlined capital fonts. Inverted delta indicates the site of the T-DNA insertion in the hws-2 allele. (TIF) [file pone.0185106.s001.tif]

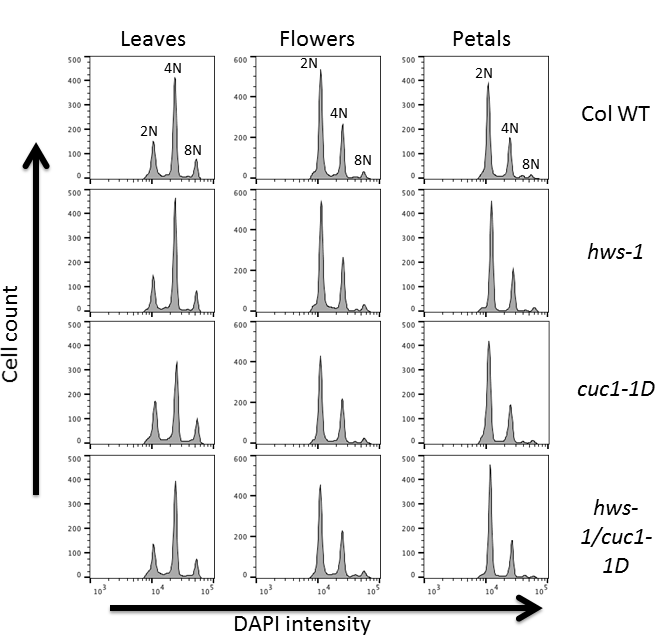

Supplement: S2 Fig — Nuclei were isolated from young leaves, flowers and petals of Arabidopsis thaliana Columbia-0, hws-1, cuc1-1D, hws-1/cuc1-1D. After DAPI staining, samples were analysed by FACS. Peaks represent cells with 2N, 4N and 8N DNA content. The X axis represents DAPI intensity and the Y axis shows cell number. (TIF) [file pone.0185106.s002.tif]

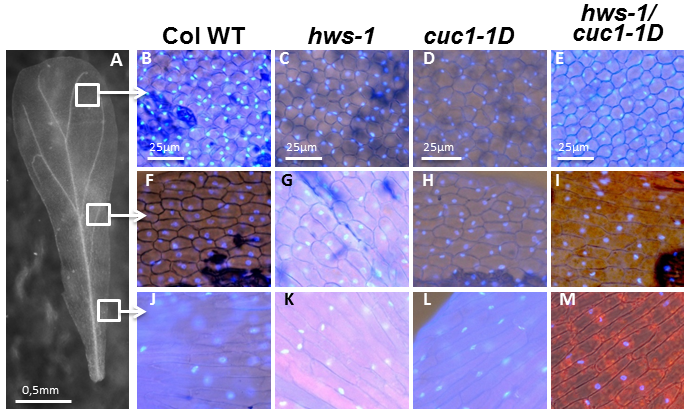

Supplement: S3 Fig — (A), Petal of Arabidopsis thaliana. DAPI stained nuclei were observed in the top part conical cells, in the middle transition area and at the bottom part of the petal (white squares) Bar = 0.5mm. (B-E), DAPI stained nuclei in upper conical cells of Arabidopsis petal from Columbia-0, hws-1, cuc1-1D, hws-1/cuc1-1D, respectively. (F-I), DAPI stained nuclei in cells of the middle transition area of Arabidopsis petal from Columbia-0, hws-1, cuc1-1D, hws-1/cuc1-1D, respectively. (J-M), DAPI stained nuclei in cells of the bottom area of Arabidopsis petal from Columbia-0, hws-1, cuc1-1D, hws-1/cuc1-1D, respectively. Scale bar: 25μm. (TIF) [file pone.0185106.s003.tif]
