# Supplementary material for: HAWAIIAN SKIRT controls size and floral organ number by modulating CUC1 and CUC2 expression
Source: PLoS One. 2017 Sep 21;12(9):e0185106. doi: 10.1371/journal.pone.0185106 (PMC5608315; doi:10.1371/journal.pone.0185106)
Supplement: S1 Table — Experiment or procedure, primer name and sequences of primers are included. (DOC) [file pone.0185106.s004.doc]

**S1 Table. Sequence of Primers used in this study.**

Experiment or procedure, primer name and sequences of primers are included.

| **Experiment or procedure:** | **Primer name:** | **Sequence 5’ to 3’:** |
| --- | --- | --- |
| Sequencing primers for *ffo1* | At3g61590 ForcDNA | GCTCTTGAGAATGGAAGCAGAAAC |
|  | At3g61590Rev | CAGACCCATTTGCTTCTTCATTGC |
|  | SSLPHSRev | GTGCCACTACTCGCGAAAACCTCG |
|  | SSLPHSFor | GAGAGAGGCTTGTGATTGTCGGAG |
|  | HS5’endutrfor | CCGCATTTCCTCTCGCTAATCTATTC |
|  | HSmap3rev | GGTATTTACTCTGGCAACCAGAG |
| Primers to identify *hws-1*, qRT-PCR analysis, and *in situ* probe | SSLPHSRev | GTGCCACTACTCGCGAAAACCTCG |
|  | SSLPHSFor | GAGAGAGGCTTGTGATTGTCGGAG |
| Primers to identify *ffo1* | Ffo1REVWTmod | TGTAGAGATGAATAGAGAGTTCC |
|  | ffo1REVMUTmod | CTGTAGAGATGAATAGAGAGTTCT |
|  | Ffo1FORWTmod | GCAAGTTCCAGGGAATTTCTTCCAGTGG |
|  | Ffo1FORMUTmod | GAAGCAAGTTCCAGGGAATTTCTTCCAGTGA |
| Primers to Map *prb1* | CER452410F | TTCGTCGACTTCTCTCACACA |
|  | CER452410R | TTTATTCACCAACAACCCAGA |
|  | 473863F | GTCACCACATTAATTCCAAGA |
|  | 473863R | TGGTAACACCCTCTTTCTCCA |
|  | 470642F | ATCTGACGTGGACGGAATCT |
|  | 470642R | GAGTGTAGTGGCCGTTGGAT |
|  | CER455359F | GGTTGCGCTAACCAGAAA |
|  | CER455359R | ATTCGTCGCCCAAGTATTGT |
|  | nga162-F | CATGCAATTTGCATCTGAGG |
|  | nga162-R | | CTCTGTCACTCTTTTCCTCTGG | | --- | |
|  | CER477120F | GCTGTTGAAACTAACACCAT |
|  | CER477120R | GTTCAACCATATGAGCTCTG |
| **Experiment or procedure:** | **Primer name:** | **Sequence 5’ to 3’:** |
|  | CER457003F | TGAGGTCGTTTAATGTAGAT |
|  | CER457003R | CCTGAACCTTTCTAGTTACC |
|  | CIW11F | CCCCGAGTTGAGGTATT |
|  | CIW11R | GAAGAAATTCCTAAAGCATTC |
|  | 476627F | GCAATCACATGTTAGGCGTAC |
|  | 476627R | CTGGCCTACTAGAGAGCTCC |
|  | 477721F | CTGCGTTATAGTTTTTCATGAGG |
|  | 477721F | CTGCGTTATAGTTTTTCATGAGG |
| Sequencing primers for *prb1 (CUC1-1D)* | CUC1For | GTGCCGACAATGGATGTTGATGTG |
|  | CUC1Rev | GCATGGCGATCAGAGAGTAAACGG |
|  | CUC1-dCAPsFor | ACAAAATCACTTCTCGGGATGTAGA |
|  | CUC1-dCAPsRev | ATACTTTACCTTAGCGGAGGAGGA |
|  | CUC1-QRTF | TCTGCCGGTTCTGCAATTG |
|  | CUC1-QRTR | CATCGGTATGAGCAGCAGAGTT |
| Primers to complement *shs-1* | Cuc1PrFor | CAATTATGACGAATTTTCTCTCGCCG |
|  | Cuc1Rev | CAGGACACGTGCTCCGTCGCAAAG |
|  | CUC1PrSalFor | GGCGGTCGACAATTATGACGAATTTTC |
|  | CUC1BamHIRev | CATAGGATCCGATCAGAGAGTAAACGG |
| Primers to identify *cuc1-1D* | CUC1FORMIS | GACAAAATCACTTCTCGGGATGAAGAC |
|  | CUC1REVSCR | CAGACAAACTTTACAGAGAACCCATTC |
| Primers to identify *cuc2-1D* | Cuc2-1Dforwt | AACCGAGCACGTGTCCTGTTGCTC |
|  | Cuc2-1Dformut | AACCGAGCACGTGTCCTGTTGCTC |
|  | Cuc2-1Drevunmod | GGAGGTCATAGTCGATGCAGAAGAC |
|  | Cuc2-1Dforunmod | GTGTCCAATGTACATTCCCTCTGTGTG |
|  | Cuc2-1Drevwt | CCGTAGTAGTAGTAGGGACATTGG |
|  | Cuc2-1Drevmut | CCGTAGTAGTAGTAGGGACATTGA |
| **Experiment or procedure:** | **Primer name:** | **Sequence 5’ to 3’:** |
| Primers for qPCR analyses |  |  |
| Cuc1 (and for *in situ* probe) | CUC1QRTF | TCTGCCGGTTCTGCAATTG |
|  | CUC1QRTR | CATCGGTATGAGCAGCAGAGTT |
| Cuc2 | CUC2FOR | ATGGCGGAGACAGCCAATATCTT |
|  | CUC2ENDREV | GACTTTGCGGAGAAGGTAATG |
| 164a | QMIR164abFOR | TGGAGAAGCAGGGCACGTGCA |
|  | QMIR164aREV | TGGAGAAGTTAAGTACGTGCAA |
| 164b (QMIR164abFOR)/ | QMIR164bREV | TGGTGAAGATGGGCACATGAAG |
| 164c | QMIR164cFOR | TGGAGAAGCAGGGCACGTGCG |
|  | QMIR164cREV | AGGGGGAGAGACACGTGTTGG |
| Tub4 | Tub4QRTFOR | CTGTTTCCGTACCCTCAAGC |
|  | Tub4QRTREV | AGGGAAACGAAGACAGCAAG |
| 164b construct | Comp164bFOr | GTAGCATGTTCATGGGTCAGCATGC |
|  | Comp164brev | CGAACAAGGTGGTAACCAGCAAGAG |
|  | Comp164bForBamHI | CTACGGATCCGTAGCATGTTCATGGG |
|  | Comp164revSacI | GACTGAGCTCCGAACAAGGTGGTAAC |
| Mutagenesis of 164b | 164b-c35t-for | GAGCAAGATGGAGAAGTAGGGCACGTGCATTAC |
|  | 164b-c35t-rev | GTAATGCACGTGCCCTACTTCTCCATCTTGCTC |
| Mutagenesis of CUC1 to produce a silent version | Prb1S-silFor | GCGACGGAGCACGTGTCTTGTTTCTCCAATAACTC |
|  | Prb1S-silrev | GAGTTATTGGAGAAACAAGACACGTGCTCCGTCGC |
